# Clinical outcomes of deferred revascularisation using fractional flow reserve in patients with and without diabetes mellitus

**DOI:** 10.1186/s12933-016-0417-2

**Published:** 2016-07-19

**Authors:** Mark W. Kennedy, Eliza Kaplan, Rik S. Hermanides, Enrico Fabris, Veemal Hemradj, Petra C. Koopmans, Jan-Hank E. Dambrink, A. T. Marcel Gosselink, Arnoud W. J. van‘t Hof, Jan Paul Ottervanger, Vincent Roolvink, Wouter S. Remkes, Aize van der Sluis, Harry Suryapranata, Elvin Kedhi

**Affiliations:** Isala Hartcentrum, Docter Van Heesweg 2, Zwolle, The Netherlands; Diagram CRO, Zwolle, The Netherlands

**Keywords:** Diabetes mellitus, Fractional flow reserve, Deferred revascularisation, Target lesion failure, Target lesion revascularisation

## Abstract

**Objective:**

Deferred revascularisation based upon fractional flow reserve (FFR >0.80) is associated with a low incidence of target lesion failure (TLF). Whether deferred revascularisation is also as safe in diabetes mellitus (DM) patients is unknown.

**Methods:**

All DM patients and the next consecutive Non-DM patients who underwent a FFR-assessment between 1/01/2010 and 31/12/2013 were included, and followed until 1/07/2015. Patients with lesions FFR >0.80 were analysed according to the presence vs. absence of DM, while patients who underwent index revascularisation in FFR-assessed or other lesions were excluded. The primary endpoint was the incidence of TLF; a composite of target lesion revascularisation (TLR) and target vessel myocardial infarction (TVMI).

**Results:**

A total of 250 patients (122 DM, 128 non-DM) who underwent deferred revascularisation of all lesions (FFR >0.80) were compared. At a mean follow up of 39.8 ± 16.3 months, DM patients compared to non-DM had a higher TLF rate, 18.1 vs 7.5 %, logrank p ≤ 0.01, Cox regression-adjusted HR 3.65 (95 % CI 1.40–9.53, p < 0.01), which was largely driven by a higher incidence of TLR (17.2 vs. 7.5 %, HR 3.52, 95 % CI 1.34–9.30, p = 0.01), whilst a non-significant but numerically higher incidence of TVMI (6.1 vs. 2.0 %, HR 3.34, 95 % CI 0.64–17.30, p = 0.15) was observed.

**Conclusions:**

This study, the largest to directly compare the clinical outcomes of FFR-guided deferred revascularisation in patients with and without DM, shows that DM patients are associated with a significantly higher TLF rate. Whether intravascular imaging, additional invasive haemodynamics or stringent risk factor modification may impact on this higher TLF rate remains unknown.

## Background

Fractional flow reserve (FFR) has an established and extensive clinical evidence base, and represents now the gold-standard invasive functional assessment of coronary lesions. Compared to angiographic assessment alone, FFR guided revascularisation results in more judicious stent placement, with subsequent cost reduction and superior clinical outcomes [1–3]. Furthermore, based on landmark trials, deferred revascularisation in haemodynamic non-significant lesions (FFR >0.80) is associated with excellent clinical outcomes and low rates of major adverse cardiac events including target lesion revascularisation (TLR) and myocardial infarction (MI) [1, 3].

However, longer-term data on deferred revascularisation primarily relates to patients with stable angina, with a low proportion of diabetes mellitus (DM) patients represented [1, 3]. DM patients have accelerated coronary atherosclerosis, an increased prevalence of microcirculatory dysfunction and a greater burden of high-risk plaque compared to non-DM patients [4–7]. Therefore, the medium to long-term outcomes of deferred revascularisation in high-risk populations with more rapidly progressive coronary atherosclerosis, such as patients with DM has not been thoroughly evaluated in previous studies. This particular study focuses on the clinical outcomes in patients with lesions which were non-haemodynamically significant (FFR >0.80) and therefore were medically treated, in DM vs non-DM patients.

## Methods

### Patient population

**Fig. 1:**
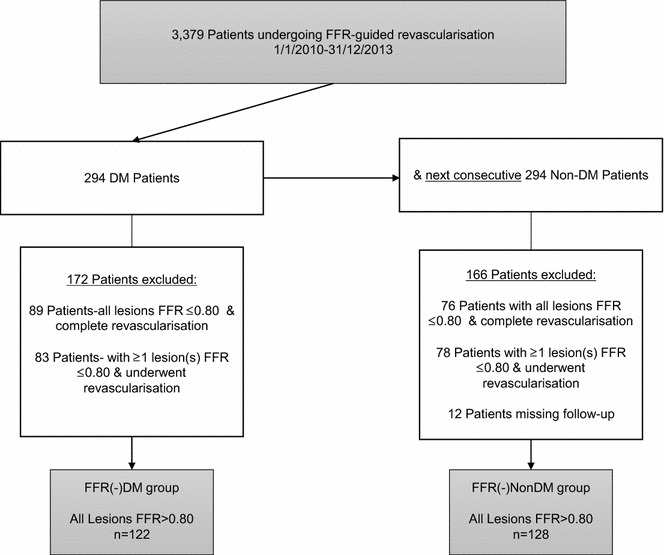
Study-flow chart. *FFR* fractional flow reserve, *DM* diabetes mellitus

In order to assess the safety and efficacy of FFR-guided deferred revascularisation, from a total of 3379 patients who underwent FFR-assessment from January 2010 until December 2013, we identified all DM patients as well as the next consecutive non-DM patients and followed these patients until 1/7/2015 (Fig. 1). In order to avoid contamination of endpoints from events occurring from revascularised lesions, we further excluded all patients where a revascularisation took place in the FFR assessed and/or other lesions, so that only patients with FFR-negative lesions were included and further analysed. As shown in Fig. 1, the two groups analysed were: DM patients with FFR-negative lesions (FFR >0.80) where revascularisation was deferred [FFR(-)DM] vs. non-DM patients with FFR-negative lesions patients where revascularisation was deferred [FFR(-)NonDM].

Baseline demographics were obtained utilising electronic medical records, as was data relating to the FFR measurement and baseline angiography. Follow up events were obtained primarily from the electronic patient record and by telephone contact with primary care physicians or direct contact with patients where required. Follow up was complete in all FFR-assessed patients. FFR assessment was systematically performed in patients with intermediate native coronary lesions ranging from 40 to 80 % diameter stenosis, where no prior non-invasive test of ischaemia was performed or when these were inconclusive. FFR was not performed for culprit lesions in MI, lesions with TIMI flow <3, or when the operator deemed a lesion to be clearly of haemodynamic significance.

The FFR was performed using a standard coronary pressure wire (PressureWire Certus, St. Jude Medical or Combowire, Volcano Corp). As per standard protocol, intravenous adenosine was infused at a rate of 140 μg/kg/min to achieve maximum hyperaemia. Both baseline FFR and maximum hyperaemic FFR values were noted for each lesion. After steady-state hyperaemia was achieved, the FFR was calculated as the ratio of mean distal intracoronary pressure measured by the pressure wire, and the mean arterial pressure measured through the coronary guiding catheter. In situations where multiple FFR measurements of a lesion were made, the lowest measurement was used as the final assessment. A cut-off value of >0.80 was taken to imply a functionally non-significant coronary stenosis and the patient underwent deferred revascularisation and continued guideline directed medical therapy.

Visual assessment of reference vessel diameter, diameter stenosis, American Heart Association/American Cardiology College (AHA/ACC) lesion type and the presence of calcification and diffuse disease were noted for all lesions by two independent interventional cardiologists. Both reviewers were blinded to the clinical outcomes. A third interventional cardiologist was used in cases where discordance arose. In addition, the syntax score (SS) was calculated, based upon the index (time of FFR-measurement) coronary angiogram, by scoring all lesions >1.5 mm with at least 50 % diameter stenosis [8]. For those patients with a prior coronary artery bypass graft (CABG), no SS was calculated. The local Institutional Review Board approved this study and waived the requirement for written consent to an institutional registry.

### Endpoints and definitions

Diabetes mellitus was defined by patient history and classified by treatment with diet, exercise, oral anti-diabetic medication or insulin. The primary endpoint was the incidence of target lesion failure (TLF), defined as a composite of target lesion revascularisation (TLR) and target vessel myocardial infarction (TVMI). Target lesion was defined as the lesion(s) in which the FFR measurement was performed, with TLR referring to revascularisation in that lesion(s). Myocardial Infarction was defined according to established guidelines [9]. Target vessel myocardial infarction (TVMI) refers to the occurrence of myocardial infarction within the vessel in which the FFR was assessed.

### Statistical methods

Continuous variables are summarised as mean ± standard deviation. Discrete variables are summarised as frequency (group percentage). Group comparisons were tested using Student’s *t* test or Mann–Whitney U test for continuous variables and Pearson’s x^2^ test for discrete data. Kaplan–Meier estimates were used to estimate survival curves, and the log-rank test was used to establish differences between groups. Cox proportional hazards multiple regression models were used to estimate differences in time to event between the two groups expressed as hazard ratio’s (HR) with 95 % confidence intervals, adjusted for several patient characteristics. In the exploratory model; gender, age, renal insufficiency, hypertension, hypercholesterolaemia, prior MI, prior PCI, prior CABG, smoking, reference vessel diameter, diameter stenosis, the presence of calcific and diffuse disease, and the absolute hyperaemic FFR value were analysed. A p value of <0.05 was considered significant. All analyses were conducted using SPSS 23 (SPSS Inc., Chicago, IL, USA).

## Results

### Baseline characteristics

As shown in Fig. 1, a total of 588 patients who underwent FFR assessment and fulfilled enrolment criteria were analysed, of which 250 patients (329 lesions) had only lesions with an FFR >0.80 and were further treated medically. Of these, 122 patients (157 lesions) and 128 patients (172 lesions) formed the FFR(-)DM group and FFR(-)NonDM groups respectively. The mean length of follow up was 39.8 ± 16.3 months (±SD).

Baseline clinical and angiographic characteristics are noted in Table 1. The average age of patients was 67.6 ± 10.2 years, however patients were older (70.4 ± 9 vs. 65.0 ± 10.7, p < 0.01) in the FFR(-)DM group. Overall, the baseline characteristics were well matched in both groups, however more patients had hypertension (95.9 vs. 80.5 %, p < 0.01), renal insufficiency (16.4 vs. 1.6 %, p < 0.01) and prior CABG (17.2 vs. 7.8 %, p = 0.02) in the FFR(-)DM group. Both the mean Syntax Score (SS) (8.71 ± 6.43 vs. 8.68 ± 5.46, p = 0.75) and the mean FFR result (0.88 ± 0.05 vs. 0.88 ±0.05, p = 0.24) was similar in both groups. Within the FFR(-)DM group, the mean HbA1c was 52.6 ± 9.1 mmol/mol.

**Table 1:** Baseline clinical characteristics

	FFR(-)DM	FFR(-)NonDM	p value
	n = 122	n = 128	
Age, years, mean ± SD	70.4 ± 9.0	65.0 ± 10.7	<0.01
Gender, male, % (n)	59 (72)	62.5 (80)	0.57
Diabetes mellitus, % (n)	100 (122)	0 (0)	
Insulin-treated, % (n)	41.8 (51)	0 (0)	
LV ejection fraction, mean ± SD	52.8 ± 10.6	51.8 ± 10.6	0.73
Multi-vessel CAD, % (n)	24.6 (30)	29.7 (38)	0.56
Family history of CAD, % (n)	32.8 (40)	32.8 (42)	0.98
Hypertension, % (n)	95.9 (117)	80.5 (103)	<0.01
Hypercholesterolemia, n % (n)	95.9 (117)	93 (119)	0.31
Current smoking, % (n)	15.6 (19)	21.1 (27)	0.26
Renal Insufficiency, % (n)	16.4 (20)	1.6 (2)	<0.01
Prior MI, % (n)	44.3 (54)	32 (41)	0.05
Prior PCI, % (n)	40.2 (49)	34.4 (44)	0.34
Prior CABG, % (n)	17.2 (21)	7.8 (10)	0.02

Renal insufficiency was defined as an estimated glomerular filtration rate, eGFR <60 mL/min

*LV* left ventricular, *CAD* coronary artery disease, *MI* myocardial infarction, *PCI* percutaneous coronary intervention, *CABG* coronary artery bypass graft

As shown in Table 2, American Heart Association/American Cardiology College (AHA/ACC) lesion type A was more frequent in the FFR(-)NonDM group (lesion level; 23.8 vs. 14.6 %, p = 0.02), whilst lesion types B2 and C were more common in the FFR(-)DM group, (34.4 vs. 22.1 %). Furthermore, patients with DM had more diffuse disease (22.3 vs. 11 %, p = 0.03). However, in both groups calcified lesions were similarly observed (19.7 vs. 18.6 %, p = 0.94) and both the reference vessel diameter (2.98 ± 0.45 vs. 3.02 ± 0.44, p = 0.41) and percentage diameter stenosis (58.8 % ± 8.3 vs. 59.1 % ± 7.0, p = 0.47) were similar in the FFR(-)DM and FFR(-)NonDM groups respectively.

**Table 2:** Baseline angiographic, FFR and lesion characteristics

	FFR(-)DM n = 122	FFR(-)NonDM n = 128	p value
Syntax score, mean ± SD	8.71 ± 6.43	8.68 ± 5.46	0.75
Low scores (0–22), % (n)	80.3 (98)	89.8 (115)	0.03
Intermediate scores (23–32), % (n)	0.8 (1)	1.6 (2)	>0.99
High scores (≥33), % (n)	1.6 (2)	0.8 (1)	0.62
Unclassified, prior CABG, % (n)	17.2 (21)	7.8 (10)	0.02
FFR performed in one lesion, % (n)	77 (94)	70.3 (90)	0.23
FFR performed in two lesions % (n)	17.2 (21)	25 (32)	0.13
FFR performed in three lesions, % (n)	5.7 (7)	4.7 (6)	0.71
FFR result, mean ± SD	0.88 ± 0.05	0.88 ± 0.05	0.24

Lesion characteristics: at lesion level	n = 157	n = 172	
*AHA/ACC lesion type classification:*			
Type A, % (n)	14.6 (23)	23.8 (41)	0.02
Type B1, % (n)	51 (80)	54.1 (93)	0.91
Type B2, % (n)	25.5 (40)	19.2 (33)	0.17
Type C, % (n)	8.9 (14)	2.9 (5)	0.07
Calcified lesion, % (n)	19.7 (31)	18.6 (32)	0.94
Diffuse disease, % (n)	22.3 (35)	11 (19)	0.03
Reference vessel diameter^a^, mean ± SD (mm)	2.98 ± 0.45	3.02 ± 0.44	0.41
Percentage of diameter stenosis^a^, mean ± SD	58.79 ± 8.3	59.07 ± 7.0	0.47

*FFR* fractional flow reserve, *AHA* American Heart Association, *ACC* American College of Cardiology, *CABG* coronary artery bypass graft

^a^Visual assessment

### Clinical outcomes

**Fig. 2:**
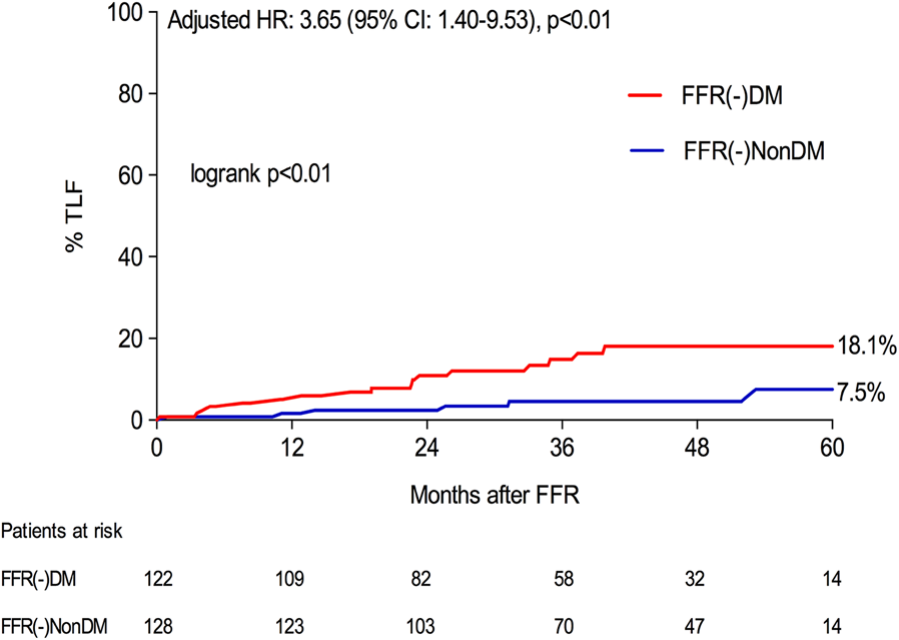
Time-to-event estimates for target lesion failure according to FFR(-)DM and FFR(-)NonDM groups. *TLF* target lesion failure, *CI* confidence interval, *HR* hazard ratio (adjusted for age)

**Fig. 3:**
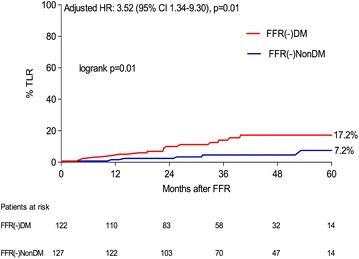
Time-to-event estimates for target lesion revascularisation according to FFR(-)DM and FFR(-)NonDM groups. *TLR* target lesion revascularisation, *CI* confidence interval, *HR* hazard ratio (adjusted for age)

**Fig. 4:**
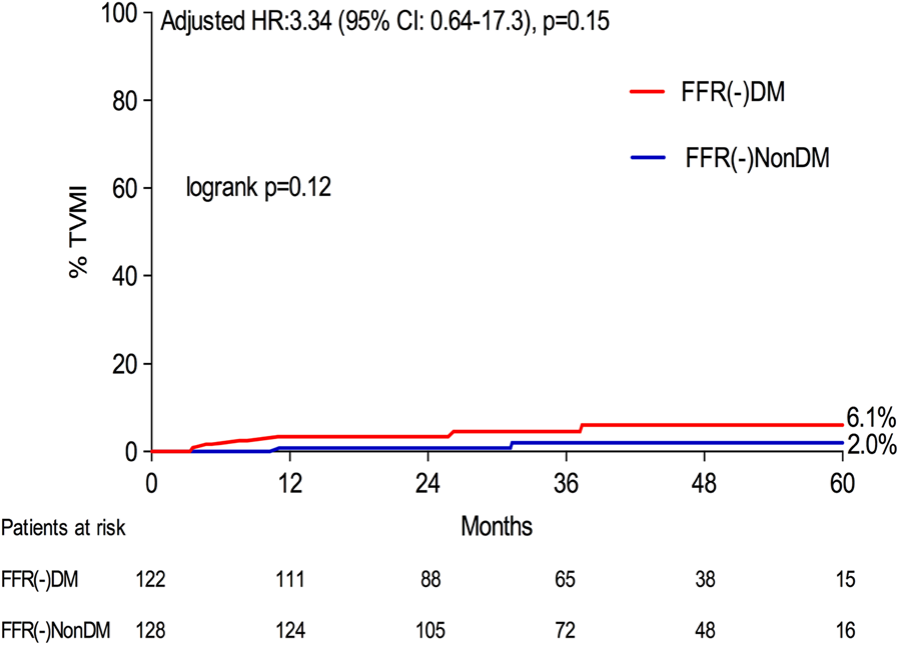
Time-to-event estimates for target vessel myocardial infarction according to FFR(-)DM and FFR(-)NonDM groups. *TVMI* target vessel myocardial infarction, *CI* confidence interval, *HR* hazard ratio (adjusted for age)

The results of the clinical outcomes are shown in Table 3 and Figs. 2 and 4 by means of Kaplan–Meier curves. The primary outcome, TLF, was observed more frequently in the FFR(-)DM group, (18.1 vs. 7.5 %, log-rank p < 0.01), Cox-regression adjusted HR 3.65 (95 % CI 1.40–9.53, p < 0.01), Fig. 2. In addition the occurrence of TLR was significantly higher in the FFR(-)DM group, adjusted HR 3.52 (95 % CI 1.34–9.30), p = 0.01, Fig. 3. In the DM group, 11/16 TLR events were as a result of TVMI or positive repeat FFR/ischaemic detection. The remaining 5/16 events were due to clear angiographic progression or unstable angina pectoris at presentation. A similar HR 3.34 (95 % CI 0.64–17.3), (p = 0.15), Table 3, Fig. 4, was also observed for TVMI, however due to lack of power, statistical significance was not reached but the trend is clear. Amongst those DM patients with TLF, HbA1c was higher (57.3 ± 14.4 vs. 51.8 ± 7.7 mmol/mol), however this did not reach statistical significance (p = 0.10).

**Table 3:** Outcome of primary endpoint and components

	FFR(-)DM n = 122	FFR(-)Non-DM n = 128	Adjusted HR (95% CI)	p value
Target lesion failure (TLF), % (n)	18.1 (17)	7.5 (6)	3.65 (1.40–9.53)	<0.01
Target lesion revascularisation (TLR), % (n)	17.2 (16)	7.5 (6)	3.52 (1.34–9.30)	0.01
Target vessel MI (TVMI), % (n)	6.1 (6)	2.0 (2)	3.34 (0.64–17.30)	0.15

Event-rates shown are Kaplan–Meier event rate estimated, % (n of events)

*HR* adjusted hazard ratio

## Discussion

The major finding of this analysis, the largest to date to directly compare the real-world clinical outcomes of haemodynamically non-significant (FFR >0.80) lesions which were treated medically in patients with versus without DM, shows that DM patients have a significantly higher risk of medium to long-term TLF. The observed higher TLF incidence in DM patients was driven primarily by efficacy events (TLR), whilst a trend for safety (TVMI) events, which did not reach statistical significance, was observed.

Presently FFR is the gold standard invasive assessment of intermediate coronary lesions, with an extensive data to support its use [1, 3, 10, 11]. The DEFER and FAME studies have shown that deferred revascularisation based on FFR guidance, is safe and associated with a favourable medium to long-term prognosis [1, 3]. However, in both these studies, a low proportion of DM patients were included. A 5-year rate of 8.9 % deferred lesion revascularisation was noted in the DEFER study (FFR >0.75), whilst in the FAME study, the 2-year rate of TLR was 3.2 % in FFR negative lesions, both findings being comparable to TLR rates in the low-risk non-DM patients in our study. Similarly, the rates of MI observed in these studies (DEFER; 5-year MI rate 0 % in deferred lesions and FAME; 0.2 % 2-year MI rate in lesions >0.80) are similar to rates observed in the non-DM patients in our study.

Moreover, since FAME and DEFER, several other real-world studies have indicated that the rate of TLR for deferred lesions may be higher than previously reported in randomised trials. Rieber et al. [12] reported in a group of 56 patients, 11 revascularisations over a 5-year period (TLR 10.7 %) and more recently, two larger studies, which included a proportion of patients with reduced LV function and also patients 1–7 days after ACS, have shown over a longer follow-up rates of TLR in deferred lesions of 20.6 % (n = 721, median follow up 48.7 months) and a 3.8 % rate of deferred lesion MI (n = 721, mean follow up 4 ± 2.3 years) [13, 14]. To date only one small single-centre study has directly examined the outcomes of deferred revascularisation in 40 DM and 96 non-DM patients, and whilst no significant difference was noted in the primary endpoint in that study, a numerically higher TLR rate (14.3 vs. 8.8 %) was noted over an average of 30 months follow-up [15]. Our results in the higher risk DM group are again comparable to the findings of these studies.

Recently, concerns regarding increased vascular resistance and reduced vasodilative capacity due to chronic hyperglycaemia in DM patients have been raised [16]. Data from prior non-invasive studies which have assessed the microcirculatory function in patients with and without DM, have shown that even in those DM patients without known coronary artery disease (CAD), the presence of an abnormal coronary flow reserve (CFR) is associated with poor outcomes, comparable to non-DM patients with known CAD [17]. Furthermore in the The Prediction of CK-MB release during successful stenting correlating with indicators of microvascular obstruction (PREDICT) trial, despite similar pre-PCI FFR values, DM patients compared to patients without, have lower CFR measurements [18].

In a study by Meuwissen et al. in patients undergoing combined FFR and CFR assessment, approximately 10 % of intermediate lesions when assessed as FFR >0.75, have an abnormal CFR defined as <2.0 and furthermore van de Hoef et al. have recently shown that in those patients in whom a FFR >0.80 is associated with an abnormal CFR (<2.0), the clinical outcomes are significantly worse than in patients with intact microcirculation [19, 20]. Recently Lee et al. [21], confirmed these findings, also showing that DM patients with low CFR and high index of microvascular resistance (IMR) have significantly elevated adverse cardiac event rates. Furthermore, whilst higher FFR values are associated with a lower incidence of adverse cardiac events in Non-DM patients with deferred revascularisation, it has recently been shown that FFR values do not differentiate future risk in DM patients [22]. As shown from these studies, FFR alone may be insufficient to ascertain the true haemodynamic significance of lesions in DM patients and therefore incorrectly lead to deferred revascularisation of lesions than may otherwise be associated with high adverse event rates. This may in part explain the results observed in the DM group in our study.

Additionally, it is known that compared to non-DM patients, atherosclerosis in diabetic patients is more often diffuse, with longer lesion length and is associated with a tendency toward negative vessel remodelling [4, 23]. Moreover, those DM patients in whom insulin treatment is required have even worse outcomes [24]. Indeed as seen in the sub-group analysis from the providing regional observations to study predictors of events in the coronary tree (PROSPECT) study; radiofrequency intravascular ultrasound assessed lesion length, plaque burden, necrotic core, and calcium content were significantly greater among non-culprit lesions in DM as compared to non-DM patients [25, 26]. Indeed, recent evidence suggests that increased levels of plasma bone morphogenetic protein-2 (BMP-2) levels are found in DM patients and correlate positively with increased plaque burden, plaque calcification and negatively correlate with lumen volume [27]. Similarly, optical coherence tomography (OCT) studies, have shown that compared to non-DM patients, non-culprit lesions in DM patients have a larger lipid index, and when associated with elevated HbA1c levels, thin-cap fibroatheroma (TCFA) and macrophage infiltration is frequently observed [7]. In addition, in a second sub-group analysis of DM patients from the PROSPECT study, non-culprit lesions in DM patients when associated with a TCFA have a five-fold increased MACE rate at 3 years, whilst those DM patients without TCFA had a 3-year MACE rate comparable to non-DM patients [28]. Thus, as shown in the above mentioned studies, DM patients more frequently carry high-risk lesions, which due to faster progression are associated with higher adverse events, despite often being angiographically mild and thus likely to be haemodynamically non-significant. Indeed, this may provide another plausible explanation for the findings of our analysis.

From the above, FFR assessment in combination with complementary haemodynamics (CFR and IMR) and also intravascular imaging may result in a more accurate deferred revascularisation and at the same time guide a more focused medical therapy strategy in DM patients. Previous studies have indicated that stringent risk factor modification in DM patients results in plaque atheroma volume regression similar to non-DM patients, and tighter glycaemic control can reduce the occurrence of recurrent ischaemic events [29–33].

### Study limitations

The present study is a single-centre, non-randomised, observational study and thus the results should be considered as hypothesis generating. Cox proportional hazards multiple regression models were used to correct for the baseline characteristics differences resulting from the lack of randomisation. Nonetheless, some residual confounding may persist despite a careful attempt to adjust for clinically relevant factors. In addition, the study took place in a region with a predominantly Caucasian population and data relating to race and ethnicity was not captured in the registry, which may impact on the generalisability of our results. As was the case in the FAME II study, neither patients nor clinicians were blinded to the FFR result [11]. Therefore, knowledge of a prior borderline FFR measurement may have influenced the subsequent rates of TLR, however considering the retrospective nature of this study this was unavoidable. Nonetheless, the majority of TLR occurred in the setting of a subsequent acute coronary syndrome and in those patients with unstable and stable angina pectoris, the majority of revascularisations only took place after repeat FFR assessment/ischaemic detection. The combined use of coronary microcirculation assessment using CFR/IMR was not performed and the morphological composition of lesions in our study was unknown and therefore their impact on our results cannot be accurately assessed.

Finally, patients included in our study may be at higher risk than those enrolled in prior randomised trials, however we believe that our results are representative of the real world outcomes of FFR-guided deferred revascularisation in patients with and without DM.

## Conclusion

DM patients in whom revascularisation was deferred based upon FFR assessment have a higher TLF rate compared to non-DM patients. Our results should be considered hypothesis generating and whether a combination of FFR with complementary haemodynamics (CFR and IMR) and intravascular imaging may result in more accurate deferred revascularisation needs to be studied in larger dedicated studies.

## Abbreviations

FFR: fractional flow reserve
PCI: percutaneous coronary intervention
CABG: coronary artery by-pass graft
MI: myocardial infarction
DM: diabetes mellitus
SS: syntax score
TLR: target lesion revascularisation
TLF: target lesion failure
TVMI: target vessel myocardial infarction
TIMI: thrombolysis in myocardial infarction
CAD: coronary artery disease
CFR: coronary flow reserve
IMR: index of microvascular resistance
BMP-2: bone morphogenetic protein-2
TCFA: thin cap fibroatheroma
